# Nutrient-Rich Foods for a Healthy Diet, Volume II

**DOI:** 10.3390/foods14152552

**Published:** 2025-07-22

**Authors:** Juristo Fonollá, Sandra González-Palacios

**Affiliations:** 1 Departamento de Nutrición y Bromatología, Universidad de Granada (UGR), 18071 Granada, Spain; 2 Centro de Investigación Biomédica en Red de Epidemiología y Salud Pública, Instituto de Salud Carlos III (CIBERESP), 28029 Madrid, Spain; sandra.gonzalezp@umh.es; 3 Instituto de Investigación Sanitaria y Biomédica de Alicante, Universidad Miguel Hernández (ISABIAL-UMH), 03010 Alicante, Spain

The growing interest in health issues in today’s society has led to worldwide concern about correct dietary habits. This requires understanding the precise composition of foods, including the percentage of macronutrients and the lesser-known bioactive compounds that give them their unique properties.

The study of the biochemistry of food and health is a dynamic and evolving field, which not only seeks to understand the composition of foods, but also to develop new functional foods and novel foods.

The success of the special issue ‘Nutrient-Rich Foods for a Healthy Diet, Volume I’ [1] encouraged MDPI to repeat the initiative with a second edition, which achieved even greater success. While the first edition comprised 7 papers, 5 original articles and 2 reviews, this second edition features an additional two publications: 5 original articles, 1 communication, 2 reviews and 1 meta-analysis.

The main objective of three of the articles in this second edition was to analyze various plants in search of bioactive compounds of interest. Thus, Gil-Martínez et al. [2] developed an ultrasound method (Sonotrode) for the extraction of compounds present in the leaves of blueberries (*Vaccinium myrtillus* L.) leaves. They tentatively identified 53 active compounds, 22 of which had not previously been detected in the leaves of this plant. The most abundant molecule was chlorogenic acid (53% of the total), and in vitro analysis demonstrated the extract’s antioxidant, antimicrobial and anticarcinogenic capacities.

The same research group presented another paper on a similar study involving blackberry fruit extract [3]. In this case, the main compounds in the extract were ursane-type terpenoids, which they believe, together with the phenolic compounds also present in the plant, may be responsible for the extract’s bioactivity. Similar properties, as well as anti-inflammatory activity, were also found in vitro.

Bolesławska et al. [4] conducted a preliminary study to determine the content of bioactive components in the fruiting bodies of four previously unstudied mushroom species: *Aleuria aurantia*, *Phallus hadriani*, *Phanus conchatus* and *Geastrum pectinatum*. The researchers analysed the antioxidant activity and polyphenol, mineral and heavy metal content. The results were similar to those of two previous studies. However, the most interesting finding was the low level of heavy metals detected in these mushrooms, making them a promising source for the development of health foods, nutraceuticals, and dietary supplements. Nevertheless, the researchers acknowledge the need for toxicological and clinical studies on the use of the fruiting bodies of these mushrooms.

In another type of study, Zaremba et al. [5] analyzed the use of selected pumpkin varieties as carriers of potassium iodide and potassium iodate at different concentrations. They hypothesised that the concentrations and form of iodine enrichment in the pumpkins would affect their antioxidant activity. The results revealed high iodine recovery in all varieties of pumpkin after drying, as well as high iodine stability during storage, particularly for potassium iodate.

In addition, this issue presents two intervention articles, one focusing on animals and the other on humans. Srisuksai et al. [6] conducted a preclinical trial involving 21 rats. They analysed the effect of crocodile oil at two doses on antioxidant activity and cognitive function in these rodents over an 8-week period. Crocodile oil is rich in monounsaturated and polyunsaturated fatty acids. The researchers found that crocodile oil significantly reduced triglyceride and free radical levels, which was more effective than olive oil. Crocodile oil had no effect on antioxidant levels in the brain. However, high doses improved memory function.

The only clinical trial published in this special issue was presented as a communication. Vázquez-Blanquiño et al. [7] analyzed the effect of consuming a capsule containing organosulphur compounds from garlic and onion daily for 36 weeks. They followed volunteers for the duration of the trial, monitoring them for any symptoms related to the virus. They found that, compared to the control group, there was a significant reduction in both the number and severity of symptoms in the treatment group, with no significant adverse effects observed. However, no significant reduction in symptom duration was detected.

As mentioned at the beginning, two reviews have also been published in this second edition of this Special Issue. The first one, by Dobrzyńska et al. [8], was based on the hypothesis that high-dose selenium supplementation has anticarcinogenic effects. This review evaluated the anticarcinogenic activity of the most commonly used selenium-rich functional products on the European market. Of the 27 articles reviewed, most of them were conducted on animals, meaning that no conclusions could be drawn regarding humans. The authors suggest that future studies should focus on epidemiological trials to help establish the daily requirement of this micronutrient, determine the optimal form of selenium for chemoprevention, and identify suitable biomarkers for monitoring disease progression.

The second review, conducted by Kolodziejczyk-Czepas [9], focused on clovamide, a molecule with similar structure as caffeic acid, and its derivatives. Clovamides are mainly present in chocolate and other cocoa-containing products in the human diet. Published reports discuss its numerous health-promoting properties. However, as in the previous review, the conclusion is that a very limited number of in vivo reports prevent the exact physiological relevance of the action of these molecules from being assessed, despite the very encouraging results of the in vitro work (antioxidant, neuroprotective and anti-neuroinflammatory properties). The author suggests that more advanced experimental models are needed to determine their bioavailability, dosage and effects in humans.

Finally, this Special Issue concludes with a systemic review and meta-analysis. Wang et al. [10] sought to determine whether dietary supplementation with linoleic acid affects blood lipid profiles. After selecting a total of 40 randomized controlled trials involving 2175 participants, they concluded that dietary intake of this fatty acid significantly decreases blood concentrations of LDL- and HDL-cholesterol. They also found that linoleic acid supplementation of more than 20 g per day is the most effective dose for improving lipid profiles, with the greatest effect observed among healthy young people with a body mass index below 30 kg/m^2^.

In short, the Special Issue ‘Nutrient-Rich Foods for a Healthy Diet’ has enabled us to conduct a comprehensive and up-to-date review of research in the field of Nutrition, thereby providing insight into the latest trends in this discipline.
